# Weak Acid Resistance A (WarA), a Novel Transcription Factor Required for Regulation of Weak-Acid Resistance and Spore-Spore Heterogeneity in Aspergillus niger

**DOI:** 10.1128/mSphere.00685-19

**Published:** 2020-01-08

**Authors:** Ivey A. Geoghegan, Malcolm Stratford, Mike Bromley, David B. Archer, Simon V. Avery

**Affiliations:** a School of Life Sciences, University of Nottingham, University Park, Nottingham, United Kingdom; b Division of Infection, Immunity & Respiratory Medicine, School of Biological Sciences, University of Manchester, Manchester, United Kingdom; Carnegie Mellon University

**Keywords:** *Aspergillus*, food spoilage, fungi, transcription factors, weak-acid resistance

## Abstract

Propionic, sorbic, and benzoic acids are organic weak acids that are widely used as food preservatives, where they play a critical role in preventing microbial growth. In this study, we uncovered new mechanisms of weak-acid resistance in molds. By screening a library of 401 transcription factor deletion strains in Aspergillus fumigatus for sorbic acid hypersensitivity, a previously uncharacterized transcription factor was identified and named weak acid resistance A (WarA). The orthologous gene in the spoilage mold Aspergillus niger was identified and deleted. WarA was required for resistance to a range of weak acids, including sorbic, propionic, and benzoic acids. A transcriptomic analysis was performed to characterize genes regulated by WarA during sorbic acid treatment in A. niger. Several genes were significantly upregulated in the wild type compared with a Δ*warA* mutant, including genes encoding putative weak-acid detoxification enzymes and transporter proteins. Among these was An14g03570, a putative ABC-type transporter which we found to be required for weak-acid resistance in A. niger. We also show that An14g03570 is a functional homologue of the Saccharomyces cerevisiae protein Pdr12p and we therefore name it PdrA. Last, resistance to sorbic acid was found to be highly heterogeneous within genetically uniform populations of ungerminated A. niger conidia, and we demonstrate that *pdrA* is a determinant of this heteroresistance. This study has identified novel mechanisms of weak-acid resistance in A. niger which could help inform and improve future food spoilage prevention strategies.

**IMPORTANCE** Weak acids are widely used as food preservatives, as they are very effective at preventing the growth of most species of bacteria and fungi. However, some species of molds can survive and grow in the concentrations of weak acid employed in food and drink products, thereby causing spoilage with resultant risks for food security and health. Current knowledge of weak-acid resistance mechanisms in these fungi is limited, especially in comparison to that in yeasts. We characterized gene functions in the spoilage mold species Aspergillus niger which are important for survival and growth in the presence of weak-acid preservatives. Such identification of weak-acid resistance mechanisms in spoilage molds will help in the design of new strategies to reduce food spoilage in the future.

## Abstract (precis)

Weak acids are widely used as food preservatives, as they are very effective at preventing the growth of most species of bacteria and fungi. However, some species of molds can survive and grow in the concentrations of weak acid employed in food and drink products, thereby causing spoilage with resultant risks for food security and health. Current knowledge of weak-acid resistance mechanisms in these fungi is limited, especially in comparison to that in yeasts. We characterized gene functions in the spoilage mold species Aspergillus niger which are important for survival and growth in the presence of weak-acid preservatives. Such identification of weak-acid resistance mechanisms in spoilage molds will help in the design of new strategies to reduce food spoilage in the future.

## INTRODUCTION

Microbiological spoilage of food and drinks is a serious threat to food security and human health. It is estimated that 25% of global food produced annually is lost due to contamination and degradation by microorganisms (1). Such spoilage also directly imperils human health, for example, due to the production of toxins by the microorganism. Preventing microbial spoilage is therefore a key to safeguarding food supply and safety. The use of chemical food preservatives to inhibit the growth of bacteria and fungi is a ubiquitous and generally effective strategy in reducing spoilage (2). Some of the most commonly used preservatives are weak organic acids such as propionic, sorbic, and benzoic acids. These are usually included in food and drink products in the form of calcium, potassium, and sodium salts, respectively.

Weak-acid preservatives are broad-spectrum antimicrobials that directly inhibit the growth of yeasts, molds, and bacteria. Although their precise mechanism of action has not yet been fully determined, it is known that weak-acid preservatives cause a reduction in cytoplasmic pH (3, 4) and inhibit nutrient uptake (5, 6). It is also known that weak acids tend to be fungistatic rather than fungicidal, especially at the levels legally permitted in food and drinks. In most cases, microbial growth is completely inhibited by the weak-acid levels used in food and drink products. However, certain species of yeasts and molds demonstrate elevated resistance to weak acids and are therefore capable of causing food spoilage (7, 8).

Weak-acid resistance can be attributed in part to the enzymatic degradation of certain acids (e.g., benzoic, sorbic, and cinnamic acids), which nullifies their antimicrobial effects. Benzoate can be catabolized through a pathway involving an initial hydroxylation step. The enzyme responsible (benzoate *para*-hydroxylase) has been found to be required for resistance to benzoic acid in Aspergillus niger and Aspergillus nidulans (9, 10). Sorbic and cinnamic acids (and certain other structurally related acids) are degraded by decarboxylation (11). In molds such as A. niger, a cluster of three genes is required for this process, encoding a transcription factor (SdrA), a decarboxylase (CdcA; formerly OhbA1 or FdcA) and a prenyltransferase (PadA) (12–15). The deletion of any of these genes reduces, but does not eliminate, resistance to sorbic acid. Thus, additional and as-yet-uncharacterized mechanisms of weak-acid resistance must operate in this mold species. Enzymatic decarboxylation of weak acids also occurs in numerous yeast species (16). However, contrary to the case in molds, deletion of the phenylacrylic acid decarboxylase gene (*PAD1*) in the yeast Saccharomyces cerevisiae does not decrease weak-acid resistance (16). Furthermore, certain spoilage yeasts do not appear to decarboxylate weak acids at all, suggesting that alternative mechanisms of resistance also operate in these species.

Mechanisms of weak-acid resistance have been best characterized in S. cerevisiae. One of the key genes required for resistance is *PDR12*, encoding an ATP-binding cassette (ABC) transporter (17). *PDR12* is required for resistance to carboxylic acids with chain lengths between 1 and 7, proposedly by mediating the efflux of weak-acid anions from the cell in an energy-dependent manner (18). *PDR12* is itself transcriptionally regulated by War1p, a Zn2Cys6 zinc finger transcription factor that binds to weak-acid response elements (WARE) in the *PDR12* promoter (19). Another transcription factor, Haa1p, is also required for resistance to weak acids in S. cerevisiae by regulating the transcription of membrane multidrug transporters (Tpo2p and Tpo3p) among other, less-well-characterized genes (20). High-throughput mutant screens have helped identify many other genes which influence weak-acid resistance in S. cerevisiae. For example, Mollapour et al. (21) reported 237 genes which were required for wild-type resistance to sorbic acid and a further 34 genes which resulted in enhanced sorbic acid resistance when deleted. A similar study, also on S. cerevisiae, revealed 650 determinants of acetic acid resistance (22). Unfortunately, there is a distinct lack of equivalent data in any other fungal species, including molds. Considering the propensity of mold fungi to cause food spoilage, understanding the genetic determinants of weak-acid resistance in these species is very important.

An additional and historically overlooked determinant of antimicrobial resistance is the phenotypic heterogeneity that exists within microbial cell populations. Phenotypic heterogeneity is a phenomenon observed within isogenic cell populations, whereby individual cells can display a markedly different phenotype despite being genetically identical. This has been recognized as an important determinant of microbial cell survival in response to antimicrobial agents and other environmental stressors (23–25). Phenotypic heterogeneity in weak-acid resistance (heteroresistance) has been found in cell populations of S. cerevisiae and the spoilage yeast Zygosaccharomyces bailii (8, 26, 27). However, there has been no investigation to date of whether weak-acid resistant subpopulations exist in populations of mold spores, although heterogeneity is known to arise in A. niger spore populations as a consequence of asynchronous conidial maturation (28). The presence of weak-acid-resistant spore subpopulations could have significant implications for spoilage control strategies and is therefore worthy of investigation.

In this study, we report the identification and characterization of a novel transcription factor (weak acid resistance A) that is required for resistance to weak-acid preservatives in A. niger and A. fumigatus. Furthermore, we identify and characterize genes that are putatively regulated by WarA, including a gene encoding a putative membrane transporter protein with similarity to S. cerevisiae Pdr12p and which, we show, mediates weak-acid resistance and heteroresistance in A. niger. These data significantly enhance our understanding of weak-acid resistance in molds and highlight both similarities and differences in weak-acid resistance strategies between yeast and mold fungi.

(This article was submitted to an online preprint archive [29].)

## RESULTS

### A screen for transcription factor deletion strains sensitive to sorbic acid identifies *warA*.

**FIG 1:**
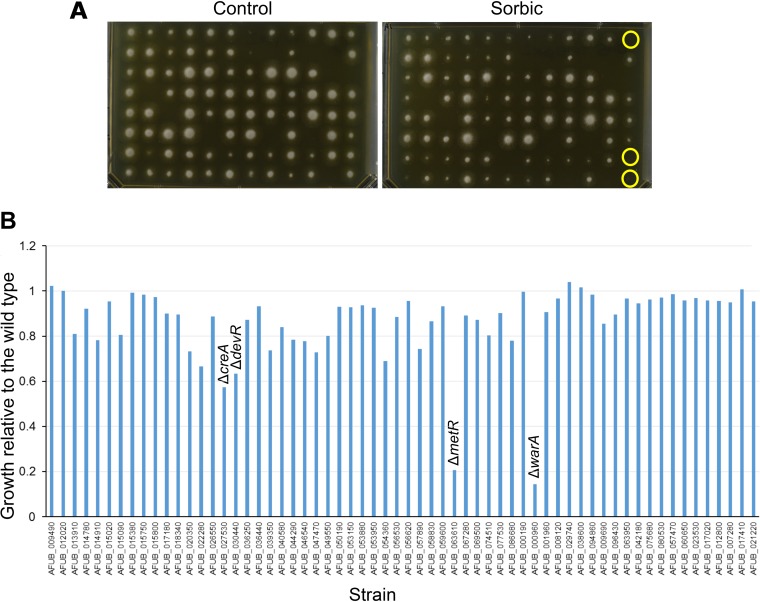
Screening of A. fumigatus deletant library. (A) Example of A. fumigatus deletant library screen. Conidial suspensions of the different deletants were arrayed in 96-well plates and transferred to growth medium using a 96-pin tool. Examples of putatively sorbic acid sensitive strains are circled in yellow. (B) Sensitivity of A. fumigatus transcription factor deletion strains to sorbic acid. Sixty-two strains were identified from the initial screen in panel A as putatively sorbic acid hypersensitive and subjected to a second round of screening, as outlined in Materials and Methods. Sensitivity to sorbic acid relative to the WT strain is shown (a value of 1 indicates identical sensitivity of the deletion strain to the WT, according to radial growth). Δ*warA* refers to the Δ*AFUB_000960* mutant.

**FIG 2:**
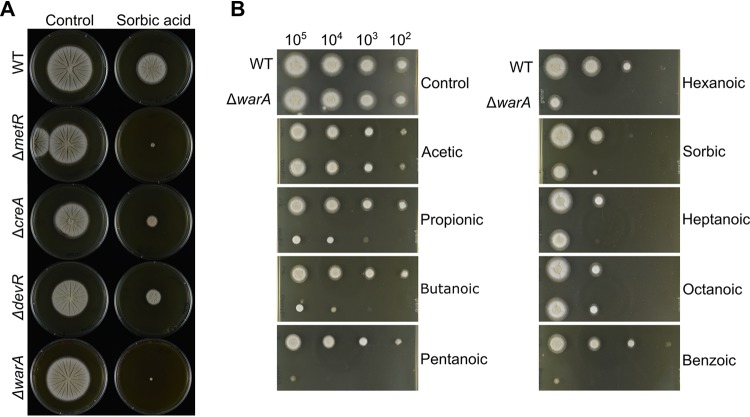
Growth of A. fumigatus transcription factor deletion strains on medium containing weak acids. (A) Radial growth of A. fumigatus transcription factor deletion strains on agar containing 0.5 mM sorbic acid. Images were captured after 3 days of growth at 37°C. (B) Radial growth of the A. fumigatus Δ*warA* mutant and wild type on agar containing weak acids. Plates were inoculated with a 10-fold dilution series of conidial suspensions; approximate numbers of conidia are indicated above the pictures. Images were captured after 2 days of growth at 28°C and are representative of 2 or 3 independent experiments. The concentrations of the acids used are given in Materials and Methods.

To find genes associated with weak-acid resistance, an Aspergillus fumigatus transcription factor deletant collection (30) was screened for sorbic acid sensitivity. Aspergillus fumigatus is not commonly associated with food spoilage, and it displays relatively high sensitivity to weak acids such as sorbic acid (14). However, deletant collections are available in A. fumigatus, unlike aspergilli associated with food spoilage. It was reasoned that transcription factors associated with weak-acid resistance in A. fumigatus may be conserved in related spoilage species such as A. niger. This resource comprised a library of 401 deletion strains of nonessential transcription factors. To determine sensitivity of the deletion strains, radial growth was compared on agar medium with and without sorbic acid (Fig. 1). This revealed two deletion strains which were highly sensitive to sorbic acid compared with the wild-type strain (Δ*metR* and Δ*AFUB_000960* mutants) (Fig. 1 and 2A). A number of other strains exhibited moderate sensitivity to sorbic acid, including the Δ*creA* (Δ*AFUB_027530*), Δ*devA* (Δ*AFUB_030440*) (Fig. 1B and 2A), Δ*rfeD* (*ΔAFUB_022280*), Δ*AFUB_020350,* and Δ*AFUB*_*054360* mutants (Fig. 1B).

MetR is a basic leucine zipper domain (bZIP)-type transcription factor mediating the transcriptional regulation of genes involved in sulfur uptake and utilization (31). Because sorbic acid is known to decrease cellular uptake of some nutrients (6, 32), it was hypothesized that sulfur limitation could be a cause of sorbic acid sensitivity in the Δ*metR* mutant strain. To test this, the sorbic acid sensitivity of the Δ*metR* mutant strain was determined in medium supplemented with the sulfur-containing amino acid methionine. This showed that the sorbic acid sensitivity of the Δ*metR* mutant strain was abolished in the presence of supplementary methionine (see Fig. S1 in the supplemental material).

10.1128/mSphere.00685-19.1 FIG S1 Radial growth of the A. fumigatus Δ*metR* mutant strain on medium containing 0.5 mM sorbic acid with or without 0.5 mM methionine. Download FIG S1, TIF file, 0.8 MB. Copyright © 2020 Geoghegan et al. 2020 Geoghegan et al. This content is distributed under the terms of the Creative Commons Attribution 4.0 International license.

*AFUB_000960* encodes a Zn2Cys6-type transcription factor which contains a fungus-specific transcription factor domain (PF11951). To further investigate the role of *AFUB_000960* in weak-acid resistance, sensitivity of the deletion strain to a range of weak acids was evaluated (Fig. 2B). The Δ*AFUB_000960* mutant strain was sensitive to propionic, butanoic, pentanoic, hexanoic, sorbic, and benzoic acids but not to acetic acid. Because of these phenotypes, *AFUB_000960* was named weak acid resistance A (*warA*).

### A WarA orthologue in A. niger is important for weak-acid resistance.

To determine whether orthologues of WarA are present in other species of fungi, a BLAST search using the WarA of A. fumigatus (*Afu*WarA) protein sequence as a query was conducted. In A. niger, this identified An08g08340, a protein with 46.2% identity and 63% similarity to the A. fumigatus WarA protein. Orthologues of *AfuwarA* were also found to be present in Penicillium and Botrytis spp.

**FIG 3:**
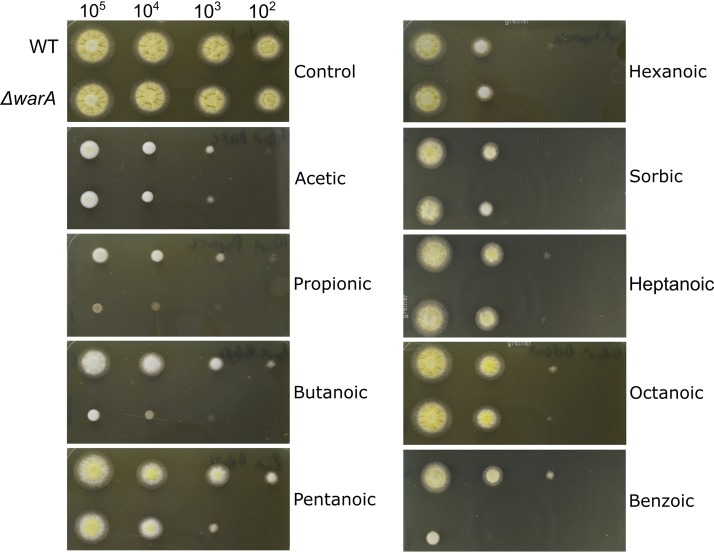
Radial growth of the A. niger Δ*warA* mutant growing on different weak acids. Plates were inoculated with a 10-fold dilution series of conidial suspensions; approximate numbers of conidia are indicated above the pictures. Images were captured after 2 days of growth at 28°C and are representative of 2 or 3 independent experiments. The concentrations of acids used are given in Materials and Methods.

Aspergillus niger is highly resistant to weak acids (especially sorbic and benzoic acids) and can readily cause food and drink spoilage. To determine whether *warA* is also required for weak-acid resistance in A. niger, *An08g08340* was deleted by a targeted gene replacement approach, with successful deletion confirmed by PCR and Southern blotting (Fig. S2). The sensitivity of the Δ*An08g08340* mutant strain to different weak acids was evaluated (Fig. 3). In contrast to the Δ*warA* mutant of A. fumigatus, the A. niger Δ*An08g08340* mutant strain demonstrated only slight sensitivity to sorbic acid, which was most apparent when individual conidia of the Δ*warA* mutant strain were spread onto medium containing sorbic acid (Fig. S3A). However, the deletant was highly sensitive to propionic, butanoic, and benzoic acids, as evaluated by radial growth on agar (Fig. 3). Determination of MICs in broth also corroborated the radial growth data; the MIC in benzoic acid was 4.5 ± 0.5 mM in the wild type (WT; *n* = 3) compared with 3.2 ± 0.2 mM in the Δ*warA* mutant (*n* = 3). The MIC in butanoic acid was 8.6 ± 0 mM in the WT (*n* = 2) and 6.9 ± 0.3 mM in the Δ*warA* mutant (*n* = 3). Resistance was restored to WT levels when *An08g08340* was reintroduced into the Δ*An08g08340* mutant strain (Fig. S4). Thus, *An08g08340* has an important role in weak-acid resistance in A. niger and was named *warA* also in this species.

10.1128/mSphere.00685-19.2 FIG S2 PCR and Southern blot confirmation of *warA* deletion. (A) Targeted gene deletion strategy. The *warA* ORF was replaced with a hygromycin resistance cassette. (B) PCR confirmation of *warA* deletion. Primer pair 1 was used to confirm deletion of *warA* ORF; deletion strains are negative, and the WT is positive. Primer pair 2 was used to confirm the integration of *HygR* at the *warA* locus. (C) Southern blotting of *warA* and *cdcA/warA* deletion strains. The gDNA of strains was digested with the HindIII restriction enzyme. Membranes were hybridized with a probe consisting of digoxigenin-UTP-labeled *HygR*. Single bands confirm single integration of the deletion cassette into the A. niger genome. The Δ*warA_12* and ΔΔ*cdcA/warA_12* transformants were used for experiments. Download FIG S2, TIF file, 0.8 MB. Copyright © 2020 Geoghegan et al. 2020 Geoghegan et al. This content is distributed under the terms of the Creative Commons Attribution 4.0 International license.

10.1128/mSphere.00685-19.3 FIG S3 (A) Growth of the Δ*warA* mutant strain conidia on medium containing sorbic acid. Plates were inoculated with ∼100 conidia and incubated for 2 days (control medium) or 3 days (medium containing 1 mM sorbic acid). (B) Radial growth of the ΔΔ*cdcA/warA* double mutant strain on medium containing 1 mM sorbic acid. Plates were inoculated with a 10-fold dilution series of conidial suspensions; approximate numbers of conidia are indicated at the top. Images were captured after 2 days of growth at 28°C and are representative of 2 or 3 independent experiments. Download FIG S3, TIF file, 1.3 MB. Copyright © 2020 Geoghegan et al. 2020 Geoghegan et al. This content is distributed under the terms of the Creative Commons Attribution 4.0 International license.

10.1128/mSphere.00685-19.4 FIG S4 Radial growth of complemented Δ*warA* strains on medium containing 2 mM benzoic acid. Plates were inoculated with a 10-fold dilution series of conidial suspensions. Approximate numbers of conidia are indicated above the pictures. Images were captured after 2 days of growth at 28°C. Two independent complemented lines are shown. Download FIG S4, TIF file, 1.1 MB. Copyright © 2020 Geoghegan et al. 2020 Geoghegan et al. This content is distributed under the terms of the Creative Commons Attribution 4.0 International license.

Sorbic acid (and structurally related acids) are known to be detoxified by decarboxylation in A. niger but not in A. fumigatus (14, 15). The decarboxylation process involves three linked genes, *cdcA*, *padA,* and *sdrA* (12, 14). CdcA is the key enzyme involved in the decarboxylation, whereas SdrA is a transcription factor regulating the expression of CdcA, and PadA synthesizes a cofactor for CdcA. It was hypothesized that the mild sorbic acid sensitivity of the A. niger Δ*warA* mutant may be due to downregulation of the *cdcA*, *padA,* or *sdrA* genes in the Δ*warA* mutant strain. To investigate the relationship between WarA and weak-acid decarboxylation, a ΔΔ*cdcA/warA* double mutant was constructed. The ΔΔ*cdcA/warA* double mutant strain was more sensitive to sorbic acid than was the Δ*cdcA* mutant strain (Fig. S3B), suggesting a *cdcA*-independent role for *warA* in resistance of A. niger to sorbic acid.

### Determination of WarA-regulated genes by transcriptomic analysis during weak-acid stress of A. niger.

**FIG 4:**
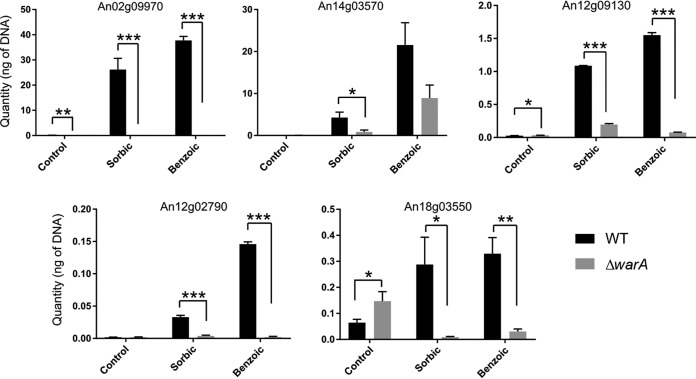
qRT-PCR of genes differentially regulated in WT and Δ*warA* mutant strains of A. niger. Transcript abundances in WT (black bars) and Δ*warA* mutant (gray bars) conidia germinated in control medium or in the presence of 1 mM sorbic acid or 1 mM benzoic acid. Error bars are standard deviation of the results from 3 technical replicates. WT and Δ*warA* mutant transcript abundances were compared by Student's *t* test (*, *P* < 0.05; **, *P* < 0.01; ***, *P* < 0.001).

The weak-acid sensitivity of the Δ*warA* mutant suggested that WarA regulates genes which are important for weak-acid resistance. Previously, genes upregulated by sorbic acid exposure in A. niger were successfully identified by exposing conidia of the wild type (WT) to sorbic acid during germination (6). In order to identify which genes are differentially regulated in the A. niger Δ*warA* mutant, RNA sequencing (RNA-seq) analysis was conducted with WT and Δ*warA* mutant conidia germinated in the presence or absence of sorbic acid. Germination of WT conidia for 1 h in the presence of 1 mM sorbic acid resulted in 3,274 differentially expressed genes (false-discovery rate [FDR]-adjusted *P* < 0.05) (1,885 upregulated and 1,467 downregulated) in comparison with conidia germinated in the absence of sorbic acid. In Δ*warA* mutant conidia, 3,442 genes were differentially expressed during germination in the presence of sorbic acid (1,885 upregulated and 1,557 downregulated), in comparison with germination without sorbic acid. Importantly, a number of genes were identified that were highly upregulated in the WT during sorbic acid exposure but not in the Δ*warA* mutant (Tables 1 and S1). This included a gene encoding benzoate *para*-hydroxylase (*bphA*), an enzyme known to be required for benzoate detoxification (9), which had a log_2_ fold change (log_2_FC) of 6.50 in the WT, compared with a log_2_FC of −0.51 in the Δ*warA* mutant. A number of uncharacterized enzymes also required *warA* for normal upregulation by sorbic acid, e.g., An12g09130 encoding a putative dienelactone hydrolase, and An12g02790 encoding a putative isoflavone reductase (log_2_FC 5.95 in the WT and log_2_FC 0.36 in the Δ*warA* mutant), as well as several genes encoding putative transporter proteins. Of particular interest among these transporters was An14g03570, an ABC-type transporter with 56% amino acid sequence similarity to S. cerevisiae Pdr12p. Pdr12p has a crucial role in weak-acid detoxification in S. cerevisiae (17, 18). To support the RNA-seq data, five of the genes showing differential expression between the WT and Δ*warA* strains were selected for quantitative reverse transcription-PCR (qRT-PCR) analysis (Fig. 4). The qRT-PCR data supported the trends in gene expression seen in the RNA-seq data set; all of the selected genes had a lower transcript abundance in the Δ*warA* mutant than in the WT during sorbic acid treatment. In addition, transcript abundances of the selected genes were compared by qRT-PCR during benzoic acid treatment. As expected, all of the genes upregulated by sorbic acid were also upregulated by benzoic acid and had lower transcript abundances in the Δ*warA* mutant than in the WT (Fig. 4).

**TABLE 1:** Transcriptomics data for selected genes upregulated in the WT during sorbic acid treatment and differentially expressed in the WT versus Δ*warA* mutant^*a*^

Gene_ID	Log_2_FC for^*b*^:		RPKM for^*c*^:		Function(s)^*d*^
	WT_sorbic vs WT control	Δ*warA*_sorbic vs Δ*warA* control	WT_sorbic	Δ*warA*_sorbic	
*An14g03570*	9.35	7.1	611.3	146	ABC-type transporter with similarity to S. cerevisiae Pdr12p
*An12g09130*	9.31	5.98	6,055.3	224.7	Possible dienelactone hydrolase function
*An02g09970*	8.56	4.35	1,532.7	7.3	Ortholog(s) have role in drug response, hexose transport, pathogenesis
*An13g02460*	8.24	3.08	283	0.3	Protein similar to NRPS (NRPS-like)
*An06g02170*	7.98	1.51	80.3	3	Ortholog(s) have *S*-adenosyl-Met-dependent methyltransferase activity
*An13g03170*	7.25	5.73	1,433.3	465.3	Unknown
*An12g09120*	7.14	2.87	312.3	31.7	Unknown
*An09g03500*	6.5	−0.51	226.3	4	Putative benzoate-*para*-hydroxylase; 3-hydroxybenzoate 4-hydroxylase
*An12g02790*	5.95	0.36	135.3	2.7	Isoflavone reductase-phenylcoumaran benzylic ether reductase type
*An08g07850*	5.81	3.77	11,809.3	3,248.7	Unknown
*An01g05850*	5.59	1.5	75.7	3	Thioesterase domain protein
*An08g01560*	5.22	−1.47	802.7	5.7	Ortholog(s) have role in meiotic cell cycle, regulation of TORC1 signaling
*An04g05240*	5.02	2.84	833	259.7	Unknown
*An11g04385*	4.9	2.02	74	5.7	Possible ubiquitin hydrolase
*An13g02450*	4.21	0	492.7	0	Six-hairpin glycosidase
*An08g01980*	3.31	1.71	309	74.7	Unknown
*An13g02290*	3.06	−0.95	73.3	1.7	Possible 3-dehydroshikimate dehydratase
*An09g05760*	2.74	−0.47	397.7	40	Ortholog(s) have actomyosin contractile ring, intermediate layer localization
*An18g03550*	2.45	−2.43	1,506.7	22.3	Similar to yeast Arr3 arsenate transporter
*An08g05750*	2.26	0.76	1,095	298	Unknown
*An16g00700*	0.96	−3.07	345	20.7	Has domain(s) with predicted 2Fe-2S cluster binding, oxidoreductase activity

a See Table S1 for a full list of genes.

b FC, fold change.

c RPKM, reads per kilobase per million.

d NRPS, nonribosomal peptide synthase; TORC1, target of rapamycin complex 1.

10.1128/mSphere.00685-19.9 TABLE S1 RPKM and log_2_FC values for A. niger genes (Excel file). Download Table S1, XLSX file, 0.9 MB. Copyright © 2020 Geoghegan et al. 2020 Geoghegan et al. This content is distributed under the terms of the Creative Commons Attribution 4.0 International license.

### Characterization of *An02g09970* and *An14g03570*.

**FIG 5:**
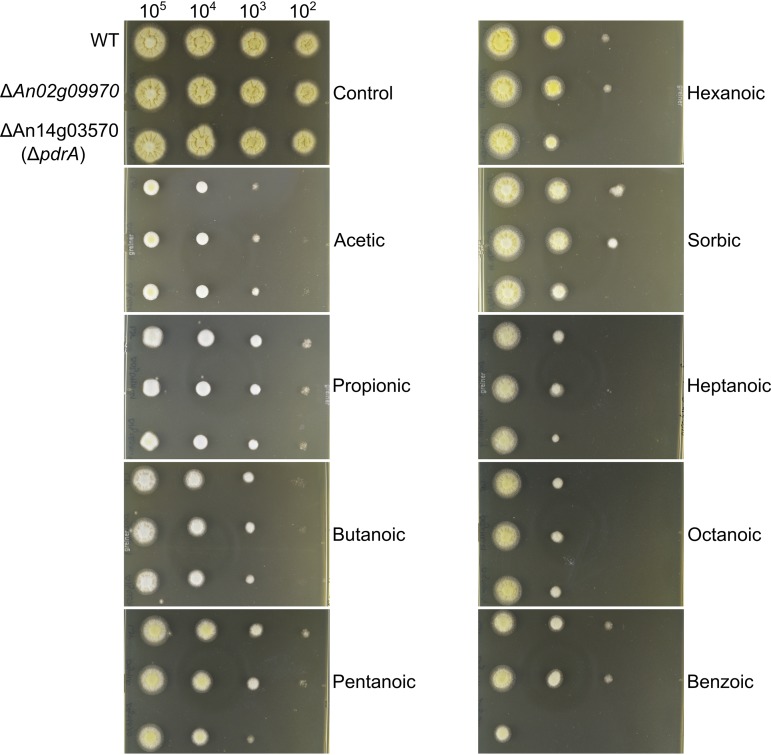
Radial growth of Δ*An02g09970* and Δ*An14g03570* (Δ*pdrA*) mutant strains growing on weak acids. Plates were inoculated with a 10-fold dilution series of conidial suspensions.

The transcriptomic analysis identified genes that are downregulated in the A. niger Δ*warA* mutant, relative to the WT strain, during weak-acid stress. These genes may therefore have a role in weak-acid resistance. To investigate this, two genes of interest (*An02g09970* and *An14g03570*) were selected for further characterization. *An02g09970* encodes a putative transmembrane transporter of the major facilitator superfamily (MFS) and was selected for further investigation due to its extremely high transcript abundance during sorbic acid treatment and large disparity in transcript abundances between the WT and Δ*warA* mutant strains (log_2_FC of 8.56 in the WT versus log_2_FC of 4.35 in the Δ*warA* mutant) (Table 1). The protein also shares significant sequence similarity with Tpo2 and Tpo3, S. cerevisiae proteins involved in resistance to acetic, propionic, and benzoic acids (20). *An14g03570* encodes an ABC-type transporter with similarity to the weak-acid detoxification protein Pdr12p in S. cerevisiae, as stated above. Both genes were deleted in A. niger by a targeted gene replacement approach, and mutant genotypes were confirmed by PCR and Southern blotting (Fig. S5). The sensitivity of the constructed deletion strains to weak acids was then evaluated. The Δ*An02g09970* mutant did not exhibit altered sensitivity to any of the weak acids tested (Fig. 5). However, the Δ*An14g03570* mutant was more sensitive to sorbic, pentanoic, and benzoic acids (Fig. 5 and Table 2), and resistance was restored to WT levels when *An14g03570* was reintroduced into the Δ*An14g03570* mutant strain (Fig. S6). Because of the similarity in sequence and function between An14g03570 and Pdr12p, An14g03570 was named PdrA.

**TABLE 2:** MIC values for A. niger WT and the Δ*An14g03570* (Δ*pdrA*) mutant strain

Acid	MIC (mM) for^*a*^:	
	WT	Δ*pdrA* mutant
Benzoic	4.67 ± 0.12	3.40 ± 0.20
Pentanoic	3.70 ± 0.35	2.60 ± 0.30
Sorbic	5.00 ± 0.35	3.80 ± 0.35

a Values are averages of 3 biological replicates ± the standard deviation.

10.1128/mSphere.00685-19.5 FIG S5 PCR and Southern blotting of *An02g09970* and *An14g03570* (*pdrA*) deletion strains. (A) Targeted gene deletion strategy. The *An02g09970* and *An14g03570* ORFs were replaced with a hygromycin resistance cassette. (B) PCR confirmation of *An02g09970* deletion. Primer pair 1 was used to confirm deletion of the *An02g09970* ORF; deletion strains are negative, and the WT is positive. Primer pairs 2 and 3 were used to confirm the integration of *HygR* at the *An02g09970* locus. (C) PCR confirmation of *An14g03570* deletion. Primer pair 1 was used to confirm deletion of the *An14g03570* ORF; deletion strains are negative, and the WT is positive. Primer pairs 2 and 3 were used to confirm integration of *HygR* at the *An14g03570* locus. (D) Southern blotting of the ΔA*n02g09970* and ΔA*n14g03570* mutant strains. The gDNA of the strains was digested with the restriction enzymes XbaI (for the ΔA*n02g09970* mutant) or EcoRV (for the ΔA*n14g03570* mutant). Membranes were hybridized with a probe consisting of digoxigenin-UDP-labeled *HygR*. Single bands confirm single integration of the deletion cassette into the A. niger genome (multiple integrations apparent for the Δ*An02g09970_39* mutant). The ΔA*n14g03570_4* and Δ*An02g09970_29* transformants were used for experiments. Download FIG S5, TIF file, 1.0 MB. Copyright © 2020 Geoghegan et al. 2020 Geoghegan et al. This content is distributed under the terms of the Creative Commons Attribution 4.0 International license.

10.1128/mSphere.00685-19.6 FIG S6 Radial growth of complemented Δ*pdrA* mutant strains on medium containing 2 mM benzoic acid. Plates were inoculated with a 10-fold dilution series of conidial suspensions. Two independent complemented lines are shown. A Δ*pdrA* mutant strain containing the empty pAN7.1BAR plasmid is also shown. Download FIG S6, TIF file, 0.8 MB. Copyright © 2020 Geoghegan et al. 2020 Geoghegan et al. This content is distributed under the terms of the Creative Commons Attribution 4.0 International license.

### Complementation of the S. cerevisiae Δ*pdr12* mutant strain with PdrA (An14g03570).

**FIG 6:**
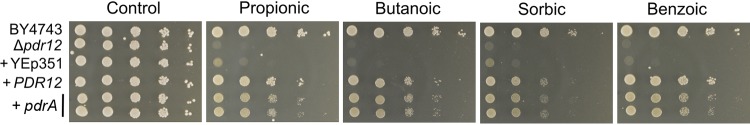
Growth of S. cerevisiae complemented strains on weak acids. Tenfold dilution series of S. cerevisiae strains (isogenic with the BY4743 wild type) were inoculated onto medium containing weak acids. The Δ*pdr12* mutant strain was transformed with either empty plasmid (+YEp351), YEp351 plasmid containing *PDR12* (+*PDR12*), or YEp351 plasmid containing the *pdrA* ORF and *PDR12* promoter and terminator (+*pdrA*). Two independent transformants of the +*pdrA* strain are shown.

The above-mentioned results showed that *pdrA* is required for resistance of A. niger to certain weak acids. Because PdrA has significant protein sequence similarity with S. cerevisiae Pdr12p (36% sequence identity, 56% similarity), it was hypothesized that these proteins could be functional homologues. To test this hypothesis, functional complementation of the S. cerevisiae Δ*pdr12* mutant strain was attempted. The cDNA sequence of A. niger *pdrA* was cloned between the S. cerevisiae *PDR12* promoter and terminator to allow for native regulation of *pdrA* in response to weak-acid stress in S. cerevisiae. The resulting plasmid (Fig. S7) was transformed into a S. cerevisiae Δ*pdr12* mutant strain. Transformants were tested for sensitivity to a range of weak acids. As hypothesized, *pdrA* could indeed functionally complement *PDR12*, as sensitivity of the Δ*pdr12* mutant strain to weak acids was largely rescued in cells transformed to express *pdrA* (Fig. 6). The resultant level of resistance was similar to that evident in Δ*pdr12* mutant cells expressing *PDR12* from the same vector backbone.

10.1128/mSphere.00685-19.7 FIG S7 Plasmid map of YEp351 containing *An14g03570* (*pdrA*). Download FIG S7, TIF file, 0.6 MB. Copyright © 2020 Geoghegan et al. 2020 Geoghegan et al. This content is distributed under the terms of the Creative Commons Attribution 4.0 International license.

### *pdrA* is a determinant of heteroresistance to sorbic acid in A. niger conidia.

**FIG 7:**
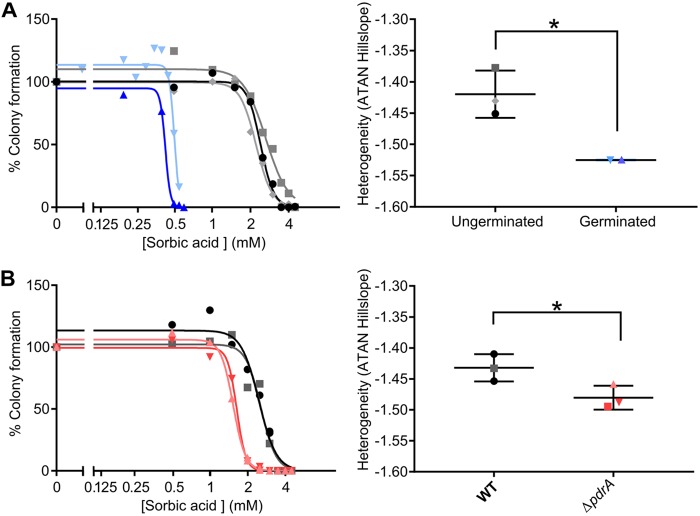
Sorbic acid dose response curves for A. niger conidia. (A) Dose-response curves of germinated (blue lines) and ungerminated (black/gray lines) WT conidia, and comparison of slope values. Dose-response curve slope values were compared with 2-way Welch’s *t* test (*P* = 0.0404,) *n* = 2 or 3. (B) Dose-response curves of WT (black/gray lines) and Δ*pdrA* mutant (pink/red lines) conidia and comparison of dose-response curve slope values, compared using a 2-way Welch’s *t* test (*P* = 0.0468), *n* = 3. Two representative independent experiments are shown in the dose-response curve. ATAN, arctangent.

Resistance to weak-acid preservatives has been found to be heterogeneous between individual cells of genetically uniform cell populations of the yeasts Zygosaccharomyces bailii and S. cerevisiae (8, 26, 27). We observed a similar phenomenon in populations of A. niger conidia, whereby small subpopulations were capable of germinating and forming colonies at high concentrations (>4 mM) of sorbic acid (Fig. 7A). Conidia harvested from colonies that grew on these high concentrations of sorbic acid did not retain increased resistance upon direct reinoculation to sorbic acid-containing medium (data not shown), suggesting that these were transient, nonheritable phenotypes, i.e., not due to genotypic variants within the population. To test whether this heteroresistance had its origin in the ungerminated conidial state, conidia were also pregerminated for 6 h before spread plating onto sorbic acid-containing medium. This showed that germinated conidia were much more susceptible to sorbic acid (Fig. 7A). Moreover, resistance to sorbic acid among pregerminated conidia was much more homogeneous than when the resistance assay commenced with ungerminated conidia, as evidenced by the gradients of the dose inhibition curves; such dose-response curves reflect heterogeneity, with shallower curves indicating greater heterogeneity (23, 33). Thus, at least some factors determining heteroresistance to sorbic acid are specific to ungerminated conidia and are lost upon germination. Given the contributions of *warA* and *pdrA* to sorbic acid resistance in A. niger, it was tested whether these genes could be determinants of heteroresistance. The dose-response curves of Δ*pdrA* and Δ*warA* mutant conidia demonstrated that *pdrA* makes a significant contribution to sorbic acid heteroresistance in A. niger conidia, whereas *warA* does not (Fig. 7B, data for Δ*warA* mutant not shown).

## DISCUSSION

This study reports the discovery of novel factors determining weak-acid resistance in molds. By screening >400 transcription factor deletion strains in A. fumigatus, we discovered a previously uncharacterized transcription factor that is required for resistance to certain weak organic acids. This transcription factor, here named weak acid resistance A (*warA*), was also found to be present in the food spoilage mold A. niger, where it also plays an important role in weak-acid resistance. However, WarA appears to mediate resistance to different weak acids in A. fumigatus and A. niger. In A. fumigatus, the Δ*warA* mutant strain is particularly sensitive to linear-chain acids 3 to 6 carbons in length, whereas in A. niger, the Δ*warA* mutant strain is most sensitive to propionic, butanoic, and benzoic acids but exhibited less sensitivity to 5- and 6-carbon acids. Such differences in acid sensitivity may reflect differences in the WarA regulon between A. niger and A. fumigatus or in the divergence in gene function within the WarA regulon.

We sought to gain insight to the WarA regulon in A. niger by conducting a comparative transcriptomics experiment between germinating WT and Δ*warA* conidia treated with sorbic acid. This approach identified several genes that appear to be regulated (either directly or indirectly) by WarA. These include a number of putative enzymes and transporter proteins, offering several candidates for future studies of weak-acid resistance mechanisms in A. niger. Among these candidates, we attempted to characterize two putative transporter protein-encoding genes. The first of these functions, An02g09970, is a transporter of the major facilitator superfamily, with sequence similarity to Tpo2p and Tpo3p in S. cerevisiae. Tpo2p and Tpo3p are transporters of the DHA1 (drug:H+ antiporter-1) family and are known to be required for resistance to acetic, propionic, and benzoic acids (20). However, the deletion of *An02g09970* did not sensitize A. niger to any of the acids tested, and so the role of this gene remains unknown. It is possible that this transporter is responsible for detoxification of other xenobiotics not tested here (if indeed it has a role in detoxification at all), or that *An02g09970* is functionally redundant with other A. niger genes. We also attempted to characterize *pdrA*, encoding an ABC-type transporter. The deletion of *pdrA* resulted in increased sensitivity to pentanoic, hexanoic, sorbic, and benzoic acids, substantiating a role for this protein in weak-acid resistance. Importantly, we were able to demonstrate that *pdrA* is a functional homologue of *PDR12* in S. cerevisiae. Pdr12p is a key protein involved in weak-acid resistance of S. cerevisiae (17), where it is thought to efflux weak-acid anions from the cytoplasm in an energy-dependent manner (18). The identification of PdrA as a functional homologue of Pdr12p in a mold species such as A. niger shows that a similar mechanism of weak-acid detoxification by active efflux may operate in yeasts and molds. Interestingly, the Δ*pdr12* mutant functional complementation experiment demonstrated that *pdrA* confers resistance to a broader range of weak acids than was suggested by the weak-acid sensitivity of the Δ*pdrA* mutant strain. For example, *pdrA* complemented the propionic acid sensitivity of the S. cerevisiae Δ*pdr12* mutant, but the A. niger Δ*pdrA* mutant strain was not more sensitive to propionic acid than was the WT. This may indicate the presence of multiple, redundant mechanisms for resistance to certain weak acids in A. niger which may not operate in S. cerevisiae.

*pdrA*, as well as several other candidate WarA-regulated genes, were all upregulated in response to both sorbic and benzoic acids. This suggests a degree of overlap between transcriptomic responses to different weak acids, as also found in S. cerevisiae (34). Thus, although we characterized the WarA regulon by comparative transcriptomics in response only to sorbic acid in the present study, it is likely that many of the differentially expressed genes would be similarly regulated in response to other weak acids. There may be relevant consensus sequences within WarA-regulated genes, although these are not apparent from promoter sequence alignments we have carried out. In S. cerevisiae, a *cis*-acting weak-acid response element (WARE) was discovered in the promoter of *PDR12* which is required for *PDR12* induction by the transcription factor War1p (19).

The regulation of WarA itself is also an outstanding question. Recent evidence in S. cerevisiae suggests that weak-acid anions bind directly to the transcription factors War1p and Haa1p, thereby regulating their DNA-binding transcriptional activation (35). However, WarA shares very little sequence homology with either War1p or Haa1p. In fact, a BLAST search of the S. cerevisiae protein database with the WarA protein sequence yields no hits at all. Nevertheless, a similar mechanism of transcription factor activation cannot be ruled out for WarA, particularly as direct ligand binding has been established for a number of Zn2Cys6 family transcription factors (of which WarA is a member), including Pdr1p, Pdr3p, Leu3p, and Put3p (36–38).

During the course of this study, experiments with sorbic acid determined that genetically uniform populations of A. niger conidia demonstrate heteroresistance to this weak acid. Phenotypic heterogeneity within microbial cell populations has been demonstrated in a number of fungi in response to environmental stresses (reviewed in reference 23); however, this is the first report of weak-acid heteroresistance in fungal conidia. Interestingly, heteroresistance was decreased within 6 h of conidial germination, suggesting that at least some factors underlying this heterogeneity are limited to ungerminated conidia and are lost upon germination. Resistance to sorbic acid was also markedly lower in germinated conidia, which has also recently found to be the case for propionic acid (39). Heteroresistance to weak acids in fungal conidia has significant implications for the food industry, because spoilage of products may occur due to contamination with just a few conidia from a highly resistant subpopulation. Thus, future spoilage control strategies may have to take into account the presence of weak-acid heteroresistance, perhaps by specifically targeting resistant subpopulations.

Conidia of the Δ*pdrA* mutant strain showed a significantly more homogeneous response to sorbic acid. Heteroresistance typically arises from gene expression heterogeneity (or noise) (40), so the present results suggest that *pdrA* could be expressed heterogeneously within conidial populations; the conidia expressing more *pdrA* are potentially able to withstand sorbic acid stress. It is also noted that the deletion of *pdrA* did not eliminate sorbic acid heteroresistance in A. niger, so it is likely that other genes also contribute.

In summary, this study markedly advances our understanding of weak-acid resistance mechanisms in A. niger. The identification of WarA as a key transcription factor involved in weak-acid resistance allowed us in turn to identify many more genes which may also be important. Further work is required to determine how all these genes may contribute to weak-acid resistance. Moreover, we demonstrated here that a key weak-acid resistance mechanism operates in both S. cerevisiae and A. niger in the form of the functionally homologous ABC transporters Pdr12p and PdrA, respectively.

## MATERIALS AND METHODS

### Strains and media.

The Aspergillus fumigatus transcription factor deletant library, derived from wild-type strain MFIG001, was constructed by homologous recombination using gene replacement cassettes and transformation methodologies described previously (30, 41). Studies in Aspergillus niger were performed in the A. niger N402 background (referred to as the A. niger WT throughout) and an A. niger Δ*cdcA* mutant strain (14). *Aspergillus* strains were cultivated on slopes of potato dextrose agar (PDA) (Sigma) for 7 days at 28°C. Conidia were harvested using 0.1% (vol/vol) Tween 80 and filtered through a 40-μm cell strainer (Fisher) before counting on a hemocytometer. Studies in S. cerevisiae used the BY4743 background and isogenic Δ*pdr12* mutant strain cultivated on YEPD agar (2% glucose, 2% Bacto peptone [Oxoid], 1% yeast extract [Oxoid], 1.5% agar) at 30°C. The S. cerevisiae strains were obtained from Euroscarf (Frankfurt). Growth assays with weak acids (see below) were performed on YEPD agar (pH 4).

### Deletant library screening and growth assays.

The first round of A. fumigatus transcription factor deletion library screening was performed in a 96-well array format. Conidial suspensions of the A. fumigatus strains were initially supplied in a 40% glycerol–0.01% phosphate-buffered saline (PBS) solution at a concentration of 4 × 10^7^ ml^−1^. These were subsequently arrayed in 96-well plates at a concentration of 4 × 10^5^ conidia ml^−1^ in 0.01% Tween 20, transferred using a 96-pin tool to Nunc OmniTray single-well plates containing YEPD agar (pH 4), and then incubated at 28°C for 2 to 3 days. Radial growth was measured using ImageJ and compared between the control medium and medium containing sorbic acid. The second round of screening was performed on 90-mm petri dishes. Plates were inoculated with 10^5^ conidia and incubated at 37°C for 3 days. Radial growth was compared between the control medium (YEPD) and the same medium containing sorbic acid.

Subsequent growth assays with weak acids on solid medium were performed in *Aspergillus* spp. by inoculating YEPD agar (pH 4) supplemented with weak acids with 5 μl of conidial suspension, containing 10^5^ to 10^2^ conidia, and subsequent incubation at 28°C for 2 to 3 days. The weak-acid concentrations used for A. fumigatus were 15 mM acetic acid, 4 mM propionic acid, 1.5 mM butanoic acid, 0.75 mM pentanoic acid, 0.2 mM sorbic acid, 0.25 mM hexanoic acid, 0.5 mM benzoic acid, 0.08 mM heptanoic acid, and 0.05 mM octanoic acid. The weak-acid concentrations used for A. niger were 40 mM acetic acid, 10 mM propionic acid, 4 mM butanoic acid, 2 mM pentanoic acid, 1.5 mM sorbic acid, 2 mM hexanoic acid, 2 mM benzoic acid, 1 mM heptanoic acid, and 0.75 mM octanoic acid.

The MICs of weak acids were determined by placing 10 ml of YEPD broth (pH 4) into 30-ml McCartney bottles and inoculating with 10^4^ conidia. The bottles were incubated statically at 28°C for 28 days, and the concentration of acids required to completely inhibit visible growth was recorded. The concentrations of acids used were at 0.2 mM increments for benzoic and sorbic acids, 0.3 mM increments for pentanoic and butanoic acids, and 2 mM increments for propionic acid.

Dose-response curves were generated by harvesting conidia as stated above, diluting to 500 spores ml^−1^, and spreading 200 μl of this onto YEPD agar (pH 4) containing sorbic acid. Plates were incubated at 28°C for up to 28 days and the colonies counted. For pregerminated conidia, conidia were first inoculated into 10 ml of YEPD-Tween 80 (6.66 ml YEPD and 3.33 ml of 0.1% Tween 80) to a final concentration of 500 spores ml^−1^ and incubated statically at 28°C for 6 h before spread plating and incubation as described above. For quantitative comparison of heteroresistance, Hill slopes were fitted to plots (% viability versus log_10_[sorbic acid]) using the Prism software, and arctangent values for the slopes were calculated with Excel to estimate relative heterogeneity (a shallower slope indicating higher heterogeneity) (33, 42).

### RNA-seq and qRT-PCR.

For preparation of RNA, conidia of A. niger N402 were inoculated into 1 liter of YEPD broth (pH 4) to a final concentration of 10^6^ conidia ml^−1^ and incubated at 28°C for 1 h, with shaking at 150 rpm. For sorbic acid or benzoic acid treatments, the medium was supplemented with 1 mM sorbic or 1 mM benzoic acid for the 1-h incubation, as these concentrations inhibit conidial germination over the course of the experiment but are not lethal (∼25% of the MIC values for these acids). Conidia were harvested by filtration through a Corning vacuum filtration unit and immediately used for RNA extraction. RNA was extracted using a Norgen Biotek plant/fungi total RNA extraction kit, as per the manufacturer’s instructions.

RNA-seq analysis was performed by the University of Liverpool Centre for Genomic Research. Three biological replicates were performed for each time point under each condition. Between 463 and 1,000 ng of total RNA (depending on available material) was poly(A) treated using the NEBNext poly(A) mRNA magnetic isolation module and subsequently purified using AMPure RNA XP beads. Successful depletion of rRNA was confirmed using Qubit fluorometric quantification (Thermo Fisher) and an Agilent 2100 Bioanalyzer. All of the depleted RNA was used as input material for the NEBNext Ultra directional RNA library prep kit for Illumina. Following 15 cycles of amplification, the libraries were purified using AMPure XP beads. Each library was quantified using a Qubit fluorometer and the size distribution assessed using the Bioanalyzer. These final libraries were pooled in equimolar amounts using the Qubit and Bioanalyzer data. The quantity was assessed using a Qubit double-stranded DNA (dsDNA) high-sensitivity (HS) assay kit, while the quality and average fragment size were assessed using the high-sensitivity DNA kit on the Agilent Bioanalyzer. The RNA libraries were sequenced on an Illumina HiSeq 4000 platform with version 1 chemistry using sequencing by synthesis (SBS) technology to generate 2 × 150-bp paired-end reads. Initial processing and quality assessment of the sequence data were performed as follows. Briefly, base calling and demultiplexing of indexed reads were performed using CASAVA version 1.8.2 (Illumina). The raw FASTQ files were trimmed to remove Illumina adapter sequences using Cutadapt version 1.2.1 (43). The option “-O 3” was set, so the 3′ end of any reads which matched the adapter sequence over a stretch of at least 3 bp was trimmed off. The reads were further trimmed to remove low-quality bases, using Sickle version 1.200, with a minimum window quality score of 20. After trimming, reads shorter than 20 bp were removed. Reads were aligned to the A. niger CBS 588.13 genome sequence (http://www.aspergillusgenome.org/download/sequence/A_niger_CBS_513_88/current/A_niger_CBS_513_88_current_chromosomes.fasta.gz) using Tophat version 2.1.0 (44). The expression of each gene was calculated from the alignment files using HTseq-count (45). The raw count data were also converted into fragments per kilobase per million (FPKM) read values. The count numbers per gene were used during the subsequent differential expression analysis. All of the differential gene expression (DGE) analyses were performed in the R (version 3.3.3) environment using the DESeq2 package (46). Significantly differentially expressed genes were defined as those with an FDR-adjusted *P* value of <0.05.

For qRT-PCR analysis of gene expression, RNA was extracted as stated above. Genomic DNA was removed using the Turbo DNase-free kit (Invitrogen). cDNA was synthesized using SuperScript IV reverse transcriptase (Invitrogen) and oligo d(T)_20_ primer (Invitrogen), according to the manufacturer’s instructions. Transcripts were amplified using SYBR green master mix on an Applied Biosystems 7500 real-time PCR instrument and quantified against a standard curve of A. niger genomic DNA. The primer pairs used are listed in Table S2.

10.1128/mSphere.00685-19.10 TABLE S2 List of primers used in this study (Excel file). Download Table S2, XLSX file, 0.02 MB. Copyright © 2020 Geoghegan et al. 2020 Geoghegan et al. This content is distributed under the terms of the Creative Commons Attribution 4.0 International license.

### Gene deletion studies and complementation in A. niger.

Gene deletion studies were performed in A. niger N402, the open reading frames (ORFs) of the target genes being replaced by a hygromycin resistance cassette (Fig. S2 and S6). Gene deletion cassettes were constructed by gap repair cloning in S. cerevisiae (47). Briefly, the hygromycin resistance cassette and the regions approximately 1 kb upstream and downstream of each target gene were amplified from genomic DNA by PCR (the primers used listed in Table S2). The hygromycin resistance cassette had a 20- to 30-bp homology with the 1-kb flanking regions, and each flanking region also had 20- to 30-bp homology with the multiple-cloning site of the YEp351 plasmid. The PCR products and HindIII-linearized YEp351 plasmid were transformed into S. cerevisiae BY4743, and transformants were selected by leucine prototrophy. Successful construction of the gene deletion cassettes was confirmed by PCR. The resulting gene deletion cassettes were amplified by PCR and purified using PCR purification columns (Macherey-Nagel) to produce a final linear gene deletion cassette. All PCRs were performed using Phusion high-fidelity DNA polymerase (New England BioLabs). Production of protoplasts and their transformation were performed using standard methods (48). Transformants were selected using 200 μg ml^−1^ hygromycin (Roche) and confirmed by PCR and Southern blotting (Fig. S2 and S6), using standard methods (49).

For complementation of A. niger gene deletion strains, the genes in question (*warA* and *pdrA* [*An14g03570*]) were amplified by PCR (primers listed in Table S2) and cloned into the SbfI site of the pAN7.1BAR plasmid (Fig. S8), which contains the *BAR* gene as a selectable marker (replacing the original hygromycin resistance cassette [50] and imparting resistance to phosphinothricin). PCR amplification included ∼1 kb upstream and ∼300 bp downstream of the ORF. Transformation of the resulting plasmids was performed as described above, except that transformants were selected using 5 mg ml^−1^ dl-phosphinothricin (Carbosynth) in YDA agar (yeast nitrogen base without amino acids, including 1.7 g liter^−1^ ammonium sulfate, 10 g liter^−1^ glucose, 2.25 g liter^−1^ ammonium nitrate, and 1 M sucrose; pH was adjusted to 7.0 using Na_2_HPO_4_, solidified with 1.2% [wt/vol] agar) (51). Transformants were subjected to an additional round of selection by growth on YDA agar containing 5 mg ml^−1^ dl-phosphinothricin.

10.1128/mSphere.00685-19.8 FIG S8 Plasmid map of pAN7.1BAR. Download FIG S8, TIF file, 0.6 MB. Copyright © 2020 Geoghegan et al. 2020 Geoghegan et al. This content is distributed under the terms of the Creative Commons Attribution 4.0 International license.

### Cloning and complementation in S. cerevisiae.

Complementation studies were performed in the S. cerevisiae Δ*pdr12* mutant strain. The complementation plasmid (Fig. S7) was constructed by yeast gap repair cloning (47). Briefly, the *PDR12* promoter and terminator and PdrA ORF were amplified by PCR (primers listed in Table S2). Each amplified fragment included a 20- to 30-bp region of homology either with the YEp351 plasmid or with a neighboring fragment. The PCR products and HindIII-linearized YEp351 plasmid were transformed into the S. cerevisiae Δ*pdr12* mutant, and transformants were selected by leucine prototrophy (47). Complementation was also performed with the *PDR12* ORF as a positive control for successful complementation. The S. cerevisiae Δ*pdr12* mutant strain was also transformed with the empty YEp351 plasmid as a negative control.

### Data availability.

RNA-seq data have been deposited in GenBank under BioProject no. PRJNA594492.
