# Supplementary figures and images for: Weak Acid Resistance A (WarA), a Novel Transcription Factor Required for Regulation of Weak-Acid Resistance and Spore-Spore Heterogeneity in Aspergillus niger
Source: mSphere. 2020 Jan 8;5(1):e00685-19. doi: 10.1128/mSphere.00685-19 (PMC6952191; doi:10.1128/mSphere.00685-19)

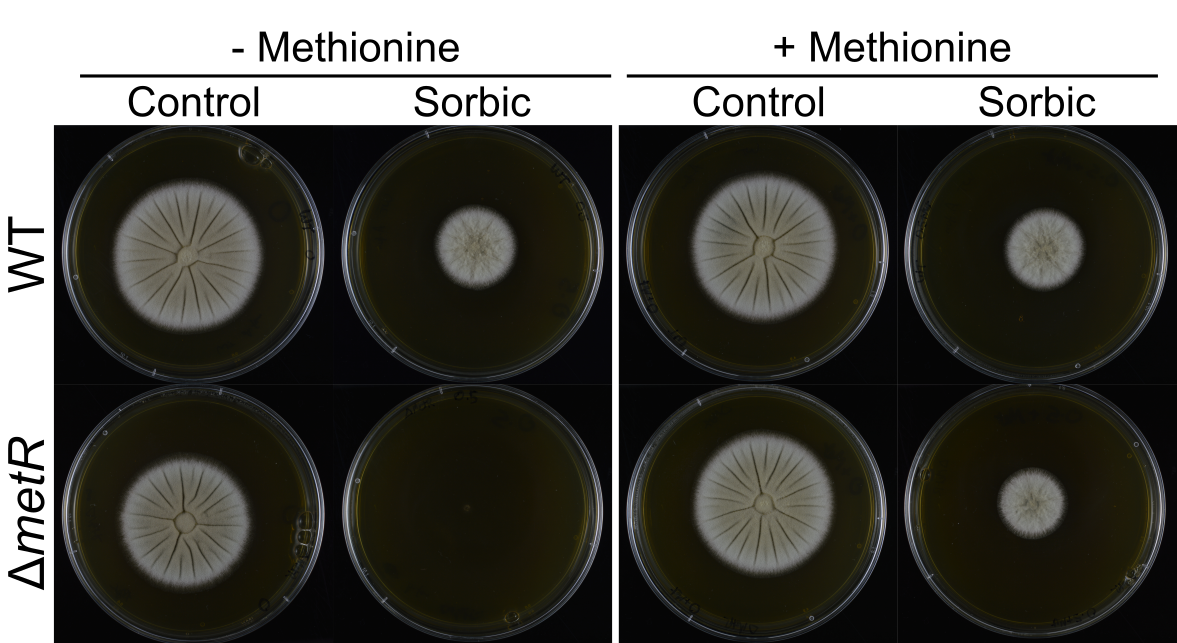

Supplement: FIG S1 [file mSphere.00685-19-sf001.tif]

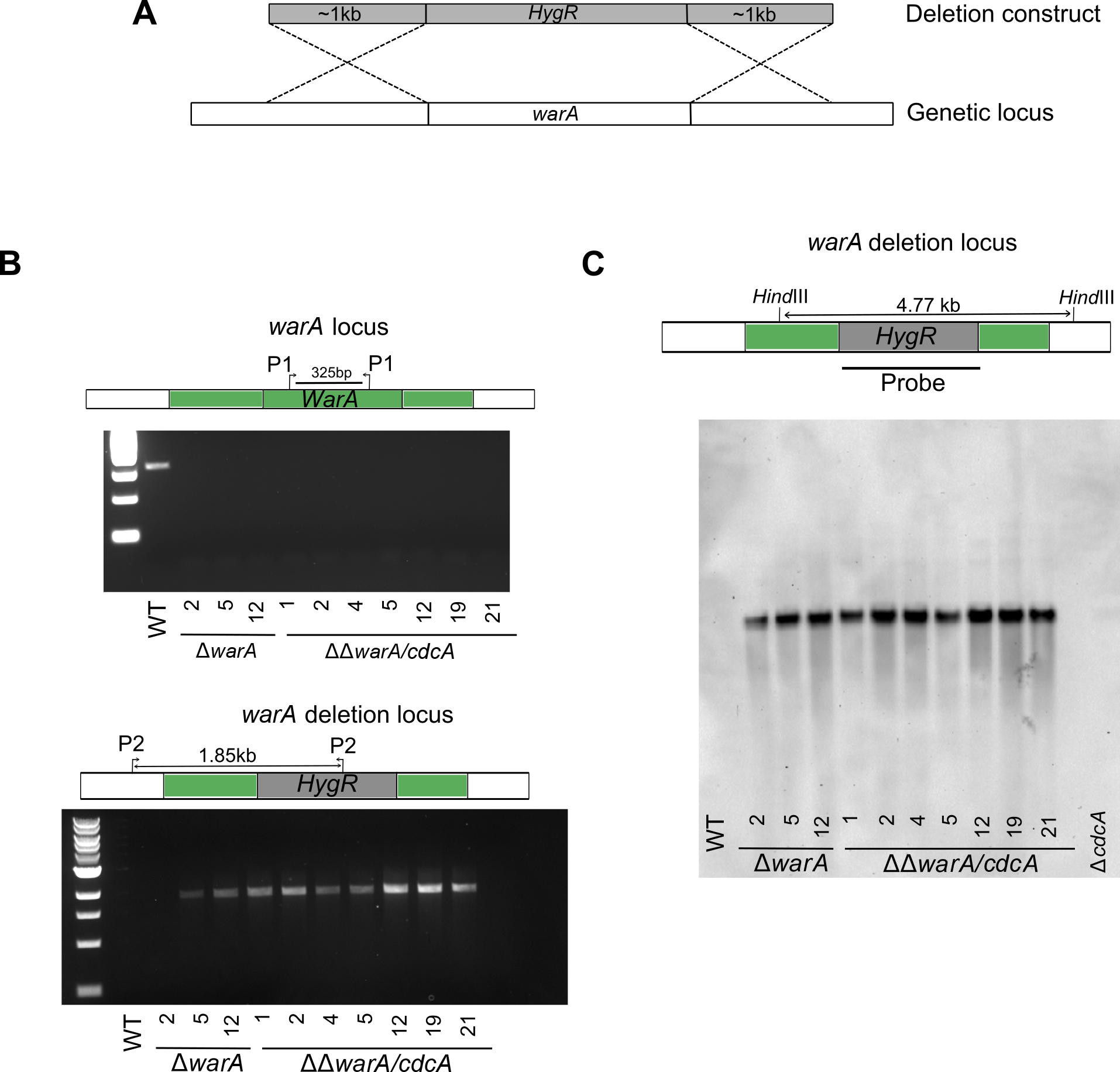

Supplement: FIG S2 [file mSphere.00685-19-sf002.tif]

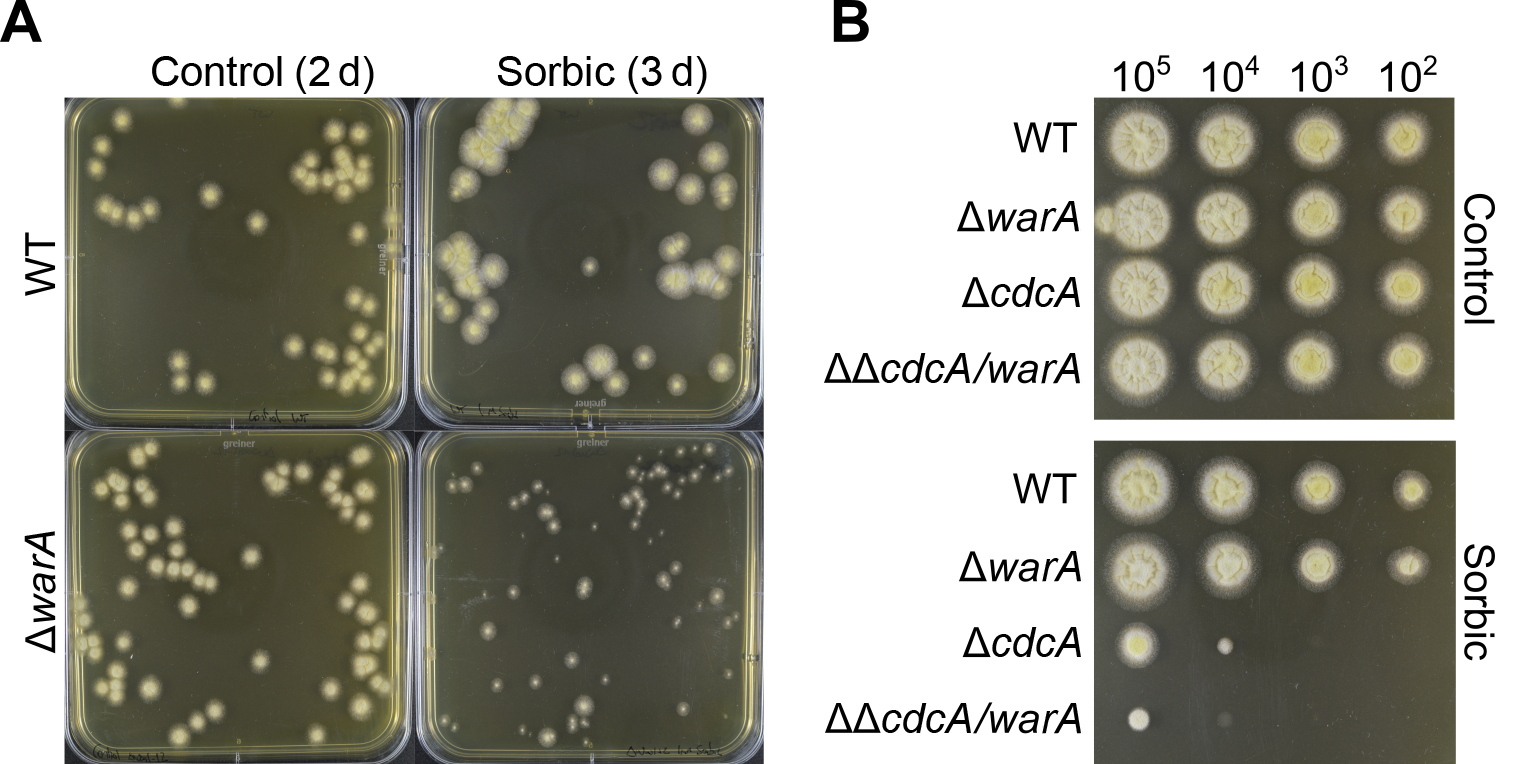

Supplement: FIG S3 [file mSphere.00685-19-sf003.tif]

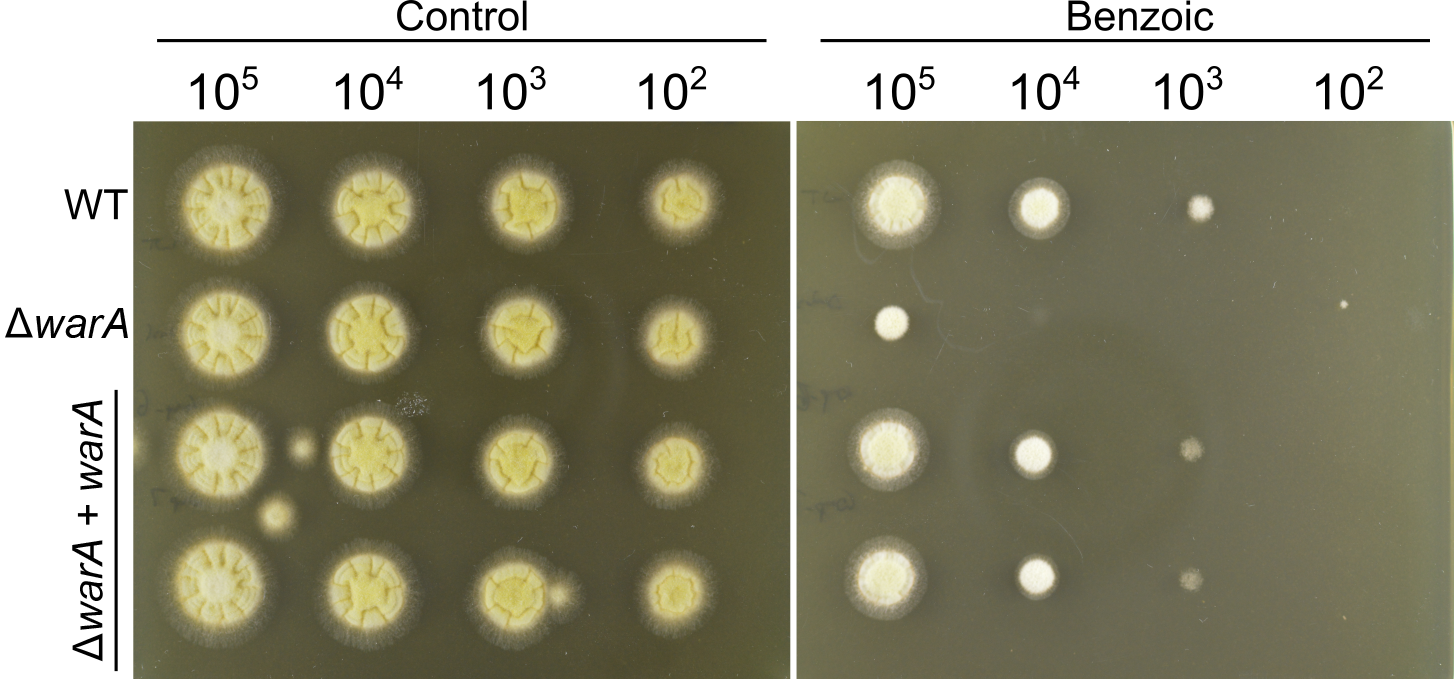

Supplement: FIG S4 [file mSphere.00685-19-sf004.tif]

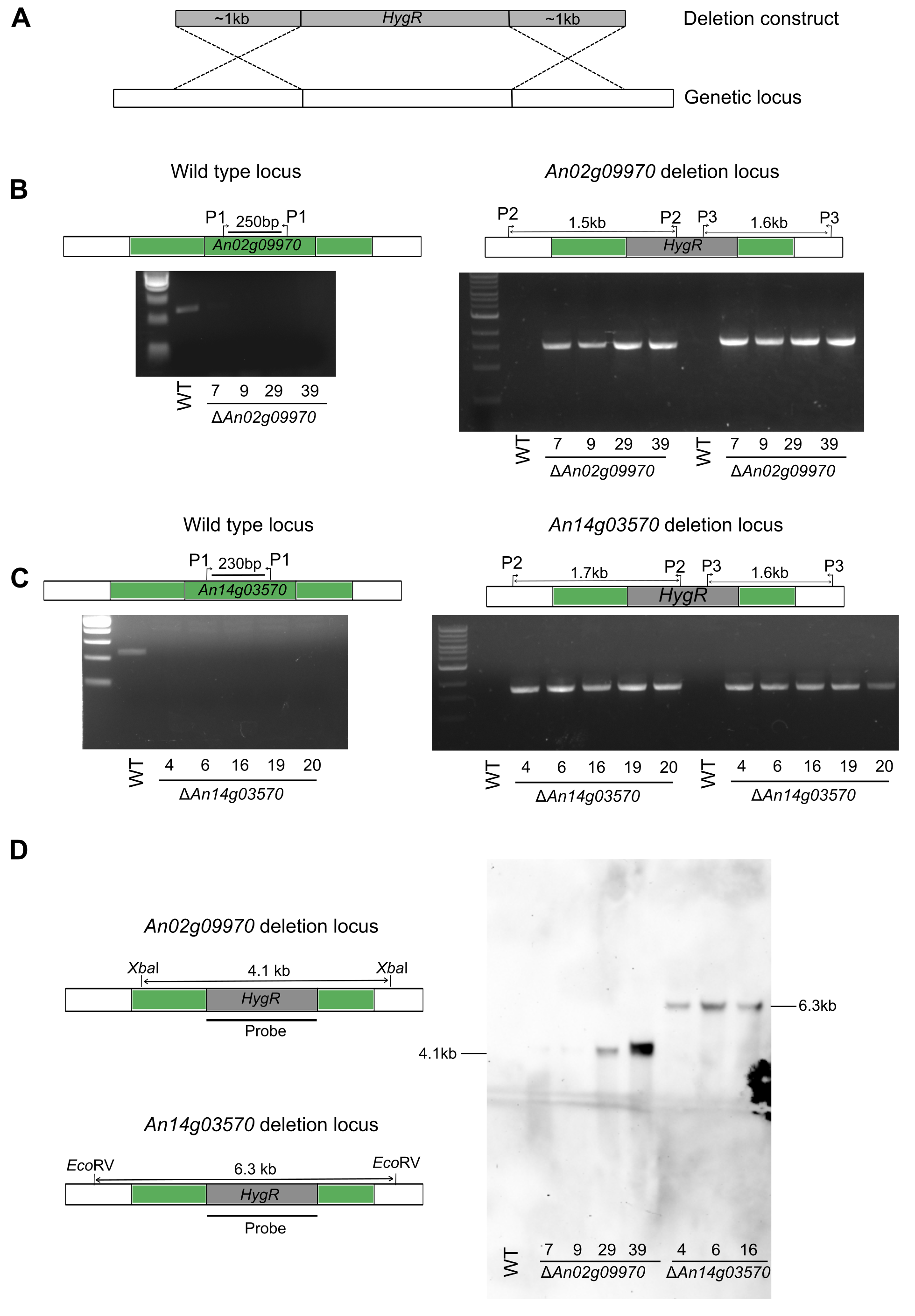

Supplement: FIG S5 [file mSphere.00685-19-sf005.tif]

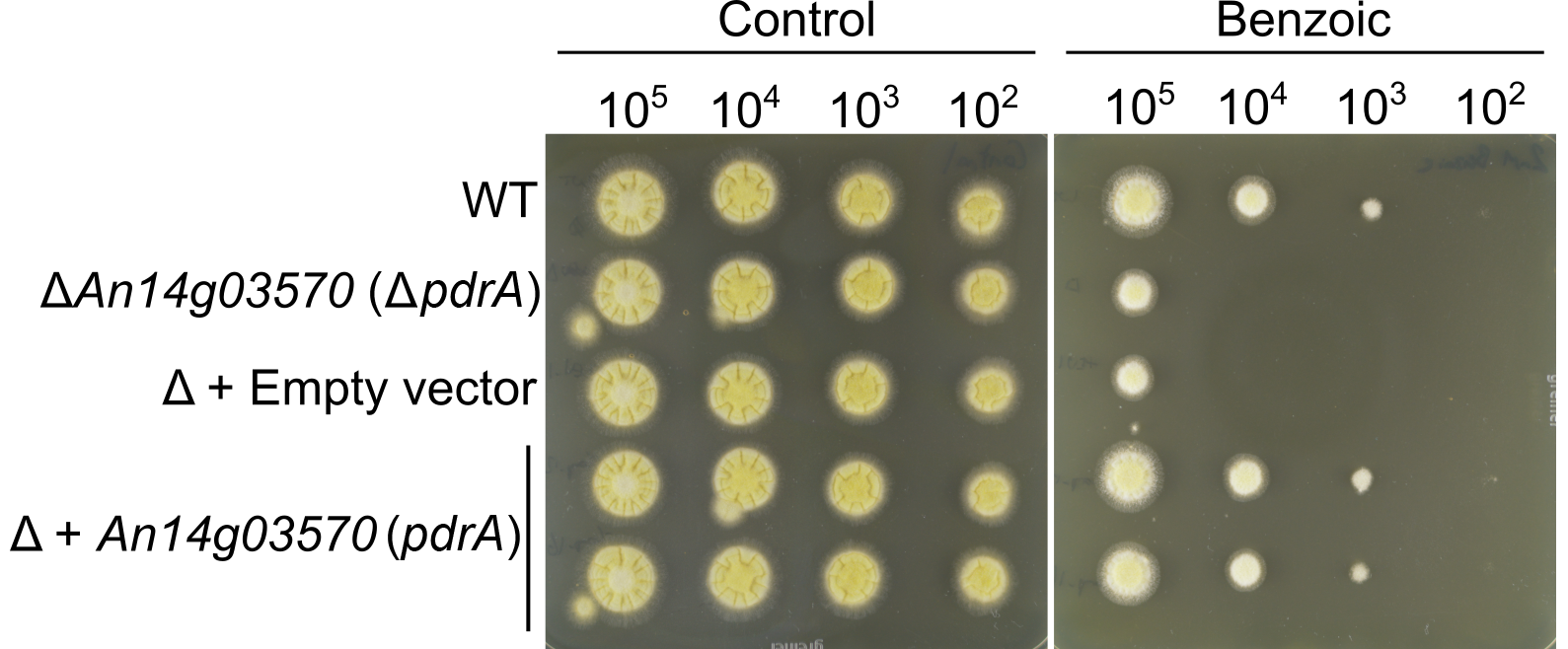

Supplement: FIG S6 [file mSphere.00685-19-sf006.tif]

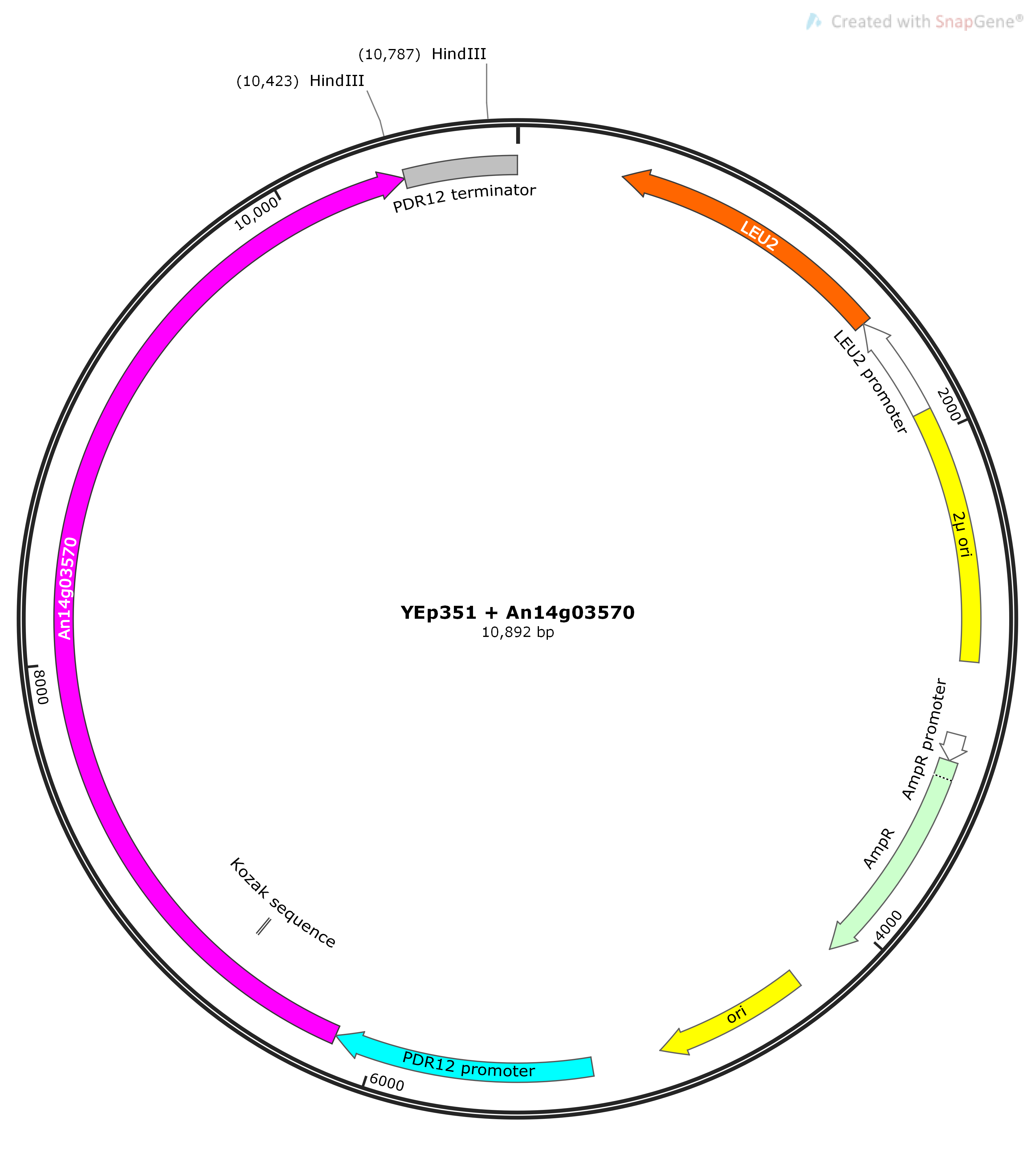

Supplement: FIG S7 [file mSphere.00685-19-sf007.tif]

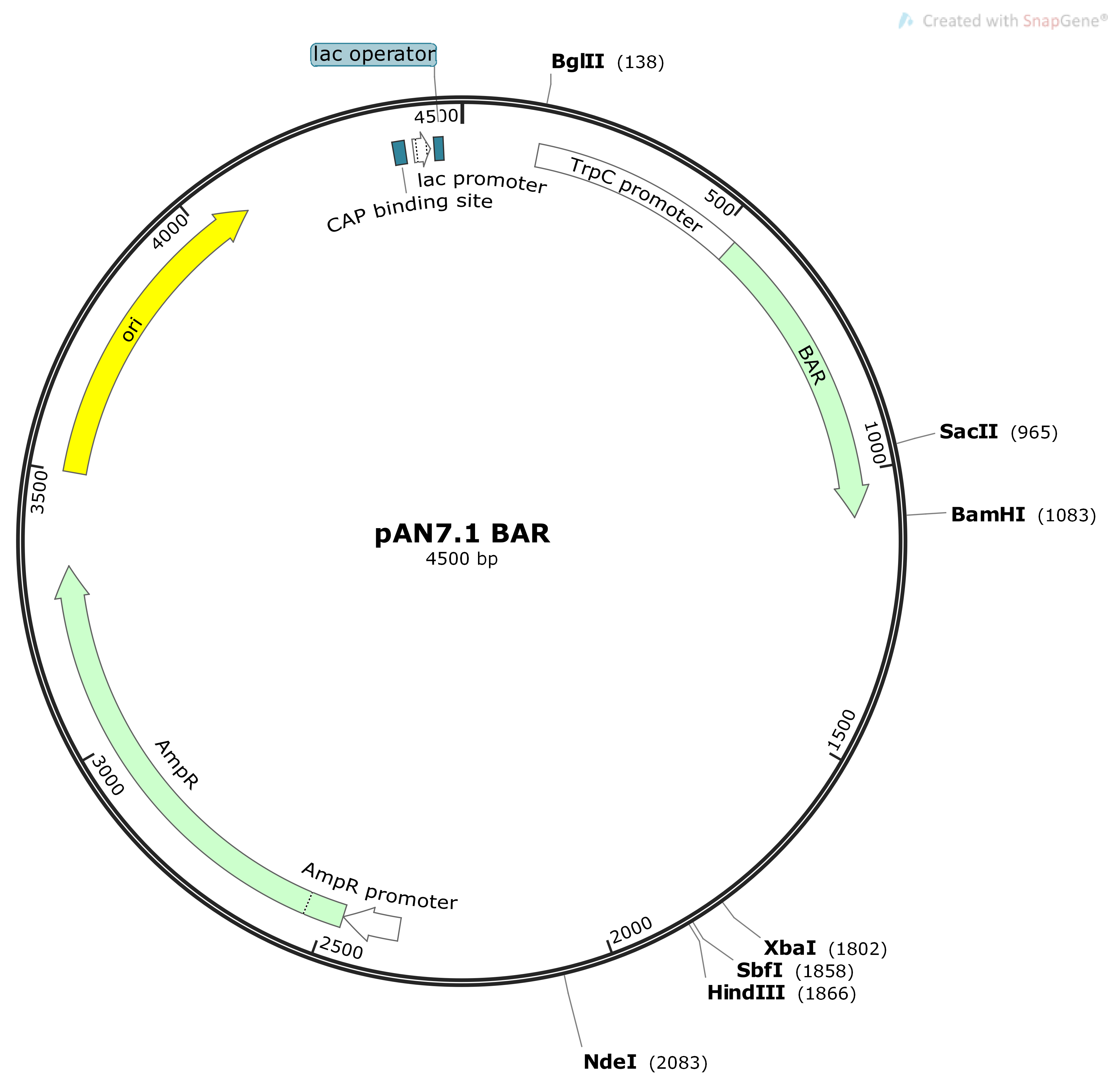

Supplement: FIG S8 [file mSphere.00685-19-sf008.tif]
